# Distribution and habitat assessments of the Slender racer, *Orientocoluber spinalis*, for the registration of nationally endangered species in the Republic of Korea

**DOI:** 10.1038/s41598-023-39018-4

**Published:** 2023-07-25

**Authors:** Daesik Park, Hojun Jeong, Jaejin Park, Il-Kook Park

**Affiliations:** Division of Science Education, Kangwon National University, Chuncheon, Gangwon 24341 Republic of Korea

**Keywords:** Ecology, Zoology, Ecology, Environmental sciences

## Abstract

Conservation assessments are essential for preserving biodiversity. However, many reptile species have not been evaluated owing to data deficiencies. The Slender racer (*Orientocoluber spinalis*) is threatened in four out of six inhabiting countries. However, despite its apparent rarity and data deficiency, the International Union for Conservation of Nature (IUCN) has classified it as a Least Concern. In this study, we combined field surveys, habitat analysis, and ecological niche models (ENMs) to identify the critical habitat characteristics of *O. spinalis*, evaluate its distribution status in the Republic of Korea, and register it as a nationally endangered species. Across the country, we found a few small populations on the mainland but large populations on the islands. *Orientocoluber spinalis* is mainly found in low-altitude ecotone habitats between grasslands and forests. Based on previous genetic and climatic studies, we propose designating it as an endangered species to conserve this species in protected areas such as national parks, and its non-isolated mainland populations can be preserved as source populations.

## Introduction

Conservation assessment is the first step in preserving biodiversity; however, many reptiles are elusive, and understanding their ecology is challenging. Notably, population status data deficiency accounted for 52% of the reasons for not being able to make global conservation assessments of reptiles^1,2^. Thus, only approximately 35% of all reptiles have been assessed by the International Union for Conservation of Nature (IUCN), and the status of unevaluated reptile species remains poorly understood^3–5^. Considering 21.1% of species are classified as threatened among 10,196 assessed reptile species^5,6^, many more reptiles could be threatened with extinction. Unassessed species without sufficient ecological data have a much higher risk of extinction than assessed species^7,8^ because they do not receive adequate attention and conservation efforts. As a result, they are now disappearing, which will be further accelerated^9,10^. For example, ignoring the general ecology of the Southern hognose snake (*Heterodon simus*) has resulted in serious population declines and local extinctions in many areas across its distribution without apparent reasons identified^11^.

Habitat destruction and degradation are the major reasons for reptile declines globally^5,12–14^ because most reptiles have a sedentary lifestyle and relatively low mobility^15,16^. Understanding the spatial ecology of reptiles and identifying their current distribution can provide key guidelines to preserve them^17^. Although field surveys effectively evaluate species habitats and distribution status, they have temporal and spatial limitations. Digital land cover maps with diverse coverage types are useful to effectively manage species habitats in large areas using location data^18^. These maps are commonly utilized to study habitat composition and change over time, enabling effective assessment and management of habitats and species^19–22^. Furthermore, spatial ecological information obtained from field surveys and land cover maps can be used to develop ecological niche models (ENMs) that reflect the habitat characteristics of species^21,22^. Ecological niche models have often been used for various conservation purposes, including the identification of potentially suitable habitats^23,24^ and habitat evaluation and management^25–27^. In particular, studies of rare species using ENMs may be highly informative for conservation planning^25,28^.

The Slender racer (*Orientocoluber spinalis*) is a rare, yet widely distributed colubrid snake, occurring across the Korean Peninsula, China, Russia, Mongolia, and Kazakhstan^29,30^. This species has a low abundance in fragmented habitats and is only observed in few protected areas in these countries^31–33^. As a result, ecological information on *O.spinalis* is globally scarce despite its wide distribution. In addition, Russia^31^, Mongolia^32^, and Kazakhstan^33,34^ even classified this species as threatened species. Nevertheless, the most recent assessment made by the IUCN on *O. spinalis* classified the species as Least Concern^35^, thus not reflecting the apparent rarity of the species across its geographic distribution. Data deficiency of this species in the Republic of Korea hampers its proper assessment. Although the observation frequency of *O*. *spinalis* is very low and decreasing^36,37^, this rare species has been just designated as potentially endangered and is not protected by the Korean Wildlife Protection and Management Act^36^. Therefore, evaluating the current conservation status of this species is urgently required, and knowledge of its spatial ecology is critical for this purpose.

Our study aimed to identify the habitat characteristics of *O. spinalis*, assess its current distribution across the Republic of Korea, and propose appropriate conservation strategies using the following five steps: (1) understanding its distribution with location data, (2) habitat analysis with field surveys and a digital land cover map, (3) evaluating suitable habitat areas using ENMs, (4) assessing the distribution status of the Korean *O. spinalis* population, and (5) providing effective strategies for the conservation. This comprehensive approach enables detailed and empirical conservation status assessments and subsequent conservation planning for the local populations in the Republic of Korea as well as other Asian countries.

## Results

### Data description

**Figure 1:**
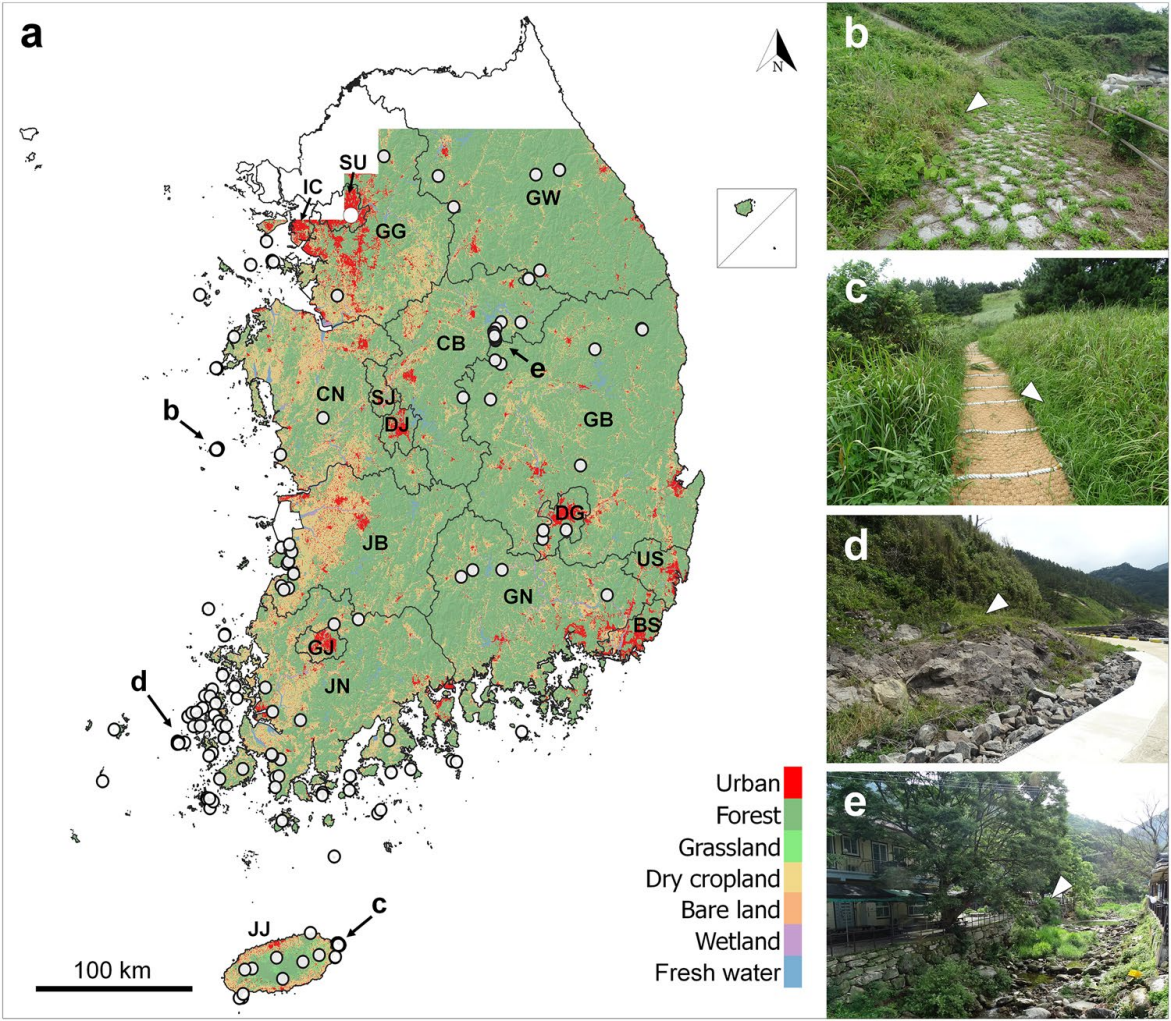
The land cover map of eight metropolitan cities and nine provinces in the Republic of Korea with 246 location data of *Orientocoluber spinalis* and four representative field survey sites (**a**). The trail on Oeyeon Island (**b**), the trail on Udo Island (**c**), bare rocks beside the road on Ui Island (**d**), and the stone wall in mountain Woraksan (**e**). White circle are location data, and triangle points to the location where the *O. spinalis* often appear. The blank in the northern part of the map was not provided detailed information on the Northern Limit Line (NLL). SU: Seoul, IC: Incheon, SJ: Sejong, DJ: Daejeon, DG: Daegu, US: Ulsan, BS: Busan, GJ: Gwangju, GG: Gyeonggi-do, GW: Gangwon-do, CB: Chungcheongbuk-do, CN: Chungcheongnam-do, GB: Gyeongsangbuk-do, GN: Gyeongsangnam-do, JB: Jeollabuk-do, JN: Jeollanam-do, and JJ: Jeju-do. This map was generated using QGIS v.3.4.7 (https://www.qgis.org). Photos by Il-Kook Park.

Initially, we collected 246 individual location data points, comprising 154 from databases and personal records and 92 from our direct field surveys (Table 1). After applying the criteria, we used 117 data points from databases and personal data and six from field collection data. Among the 246 individual location data points, 172 (69.9%) were located on the islands and 74 (30.1%) on the mainland. During the field surveys, we observed snakes mainly on Oeyeon Island (n = 51), Udo Island (n = 25), Ui Island (n = 8), and in Woraksan National Park (n = 6; Fig. 1). We filtered overlapping data points within a 1 km radius to obtain 123 data points (70 islands and 53 mainlands) for downstream analyses. Many island points were excluded from the analysis because of the high density of island populations relative to the mainland populations.

**Table 1:** Location data by 17 administrative districts and the habitable area in the ensemble model. To reduce bias, only one data was selected among the overlapped data within a radius of 1 km and used to analyze the habitat characteristics and for building the ecological niche models habitable area. The percentage indicates the habitable area relative to each partial area.

Location	Location data selection (all)			Habitable area (km^2^)		
	Island	Mainland	Total	Island	Mainland	Total
Metropolitan city						
IC	5 (5)	0 (0)	5 (5)	59.3 (34.1%)	24.0 (14.6%)	80.3 (24.6%)
DG	0 (0)	2 (2)	2 (2)	–	129.2 (16.2%)	127.5 (16.2%)
SU	0 (0)	1 (1)	1 (1)	–	70.8 (19.9%)	59.6 (19.9%)
BS	0 (0)	0 (0)	0 (0)	18.7 (52.1%)	350.1 (51.0%)	368.0 (51.1%)
DJ	0 (0)	0 (0)	0 (0)	–	7.0 (1.4%)	7.0 (1.4%)
SJ	0 (0)	0 (0)	0 (0)	–	20.4 (4.9%)	20.2 (4.9%)
GJ	0 (0)	0 (0)	0 (0)	–	162.2 (35.9%)	161.7 (35.9%)
US	0 (0)	0 (0)	0 (0)	–	301.5 (31.6%)	301.4 (31.6%)
Province						
JN	48 (68)	10 (11)	58 (79)	1111.7 (68.1%)	2764.6 (29.1%)	3738.3 (34.8%)
CN	4 (57)	2 (2)	6 (59)	93.0 (64.7%)	820.1 (11.2%)	905.0 (12.3%)
JJ	12 (41)	0 (0)	12 (41)	1081.6 (64.7%)	–	1065.9 (64.7%)
CB	0 (0)	9 (24)	9 (24)	–	114.5 (1.7%)	116.2 (1.7%)
JB	0 (0)	10 (11)	10 (11)	23.7 (59.3%)	610.5 (8.4%)	640.7 (8.7%)
GB	0 (0)	7 (8)	7 (8)	4.0 (6.0%)	656.8 (3.8%)	650.0 (3.8%)
GW	0 (0)	5 (7)	5 (7)	0.1 (66.7%)	290.3 (2.6%)	290.6 (2.6%)
GN	1 (1)	4 (4)	5 (5)	338.7 (41.3%)	1746.5 (20.0%)	2065.7 (21.9%)
GG	0 (0)	3 (4)	3 (4)	23.8 (56.0%)	393.5 (6.1%)	421.1 (6.4%)
Total	70 (172)	53 (74)	123 (246)	2754.7 (59.5%)	8462.1 (10.8%)	11,216.8 (13.5%)

### Habitat characteristics

**Figure 2:**
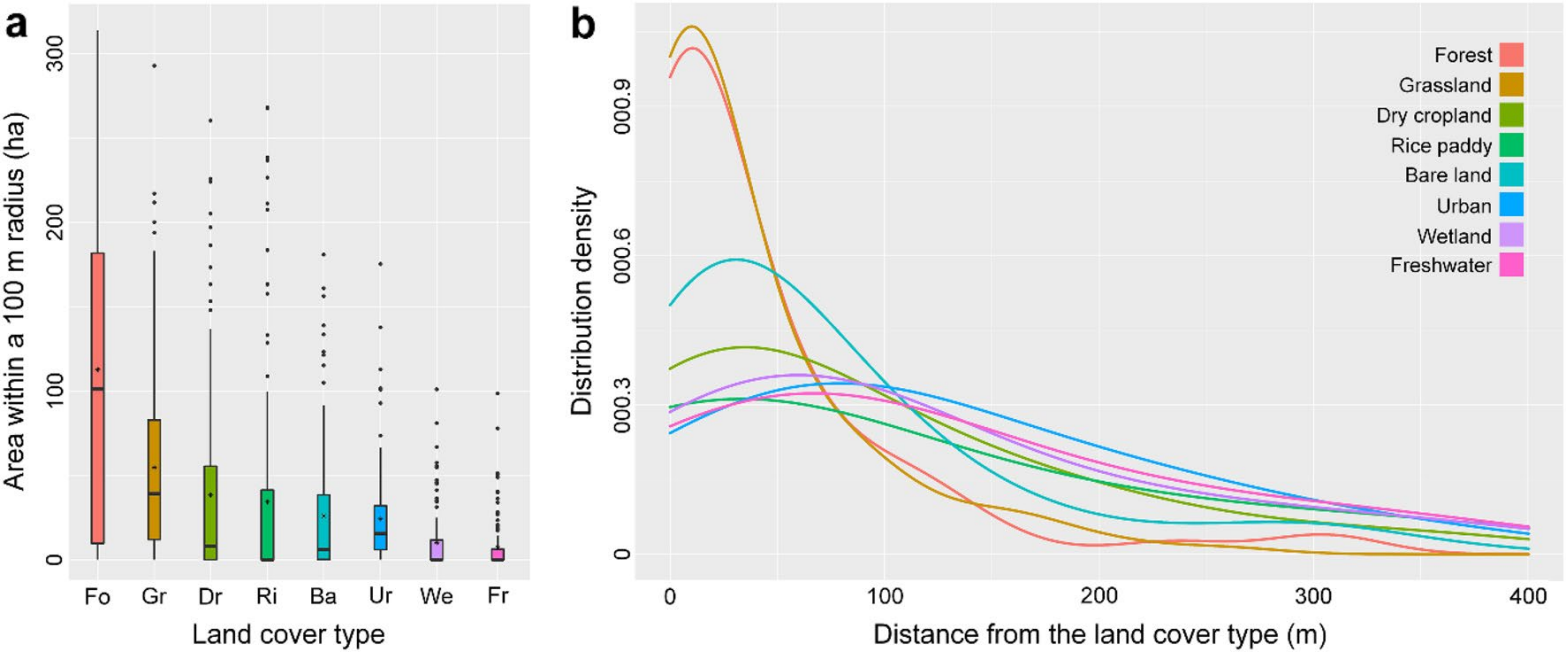
Eight land cover types of *Orientocoluber spinalis* habitat. The areas of each land cover type within a 100 m radius of *O. spinalis* location data (**a**) and distribution density map (**b**) which shows the distance from the location data to each major land cover type. This graphs were generated using the package ggplot2 (https://ggplot2.tidyverse.org) in R v.4.0.5 software package (R Core Team, https://www.r-project.org/).

The average values of the four environmental variables were as follows: altitude of 110.1 ± 159.4 m, 7.2 ± 6.1° slope, 13.0 ± 1.2 °C annual mean temperature, and 1,368.4 ± 337.6 mm annual precipitation. Habitat of *O. spinalis* (r = 100 m) was primarily composed of forest (37.7%; 1.13 ± 0.99 ha), followed by grassland (18.3%; 0.55 ± 0.57 ha), dry cropland (12.8; 0.38 ± 0.59 ha), and rice paddy (10.9%; 0.34 ± 0.66 ha) based on the individual location data (Table 2, Fig. 2). From the individual location point, grassland (49.4 ± 110.4 m) was the closest habitat to the individual location point, followed by bare land (90.1 ± 112.8 m), and forest (94.9 ± 222.0 m).

**Table 2:** Environmental variable values of 123 location data. Area indicates an area of each land cover type within a 100 m radius of each location data. Distance indicates the distances between each location and each land cover type.

Variable	Mean value ± standard deviation	
Land cover		
Forest	Area: 1.13 ± 0.99 ha (37.7%)	Distance: 94.9 ± 222.0 m
Grassland	Area: 0.55 ± 0.57 ha (18.3%)	Distance: 49.4 ± 110.4 m
Dry cropland	Area: 0.38 ± 0.59 ha (12.8%)	Distance: 144.4 ± 230.6 m
Rice paddy	Area: 0.34 ± 0.66 ha (11.5%)	Distance: 558.0 ± 1,328.7 m
Bare land	Area: 0.26 ± 0.39 ha (8.7%)	Distance: 90.1 ± 112.8 m
Urban area	Area: 0.15 ± 0.29 ha (5.2%)	Distance: 225.5 ± 434.4 m
Wetland	Area: 0.10 ± 0.19 ha (3.4%)	Distance: 485.1 ± 1,129.0 m
Freshwater body	Area: 0.07 ± 0.16 ha (2.4%)	Distance: 368.2 ± 499.2 m
Geographic variable		
Altitude	110.1 ± 159.4 m	
Slope	7.2 ± 6.1°	
Climatic variable		
Annual mean temperature	13.0 ± 1.2 °C	
Annual precipitation	1368.4 ± 337.6 mm

### Distribution characteristics

**Figure 3:**
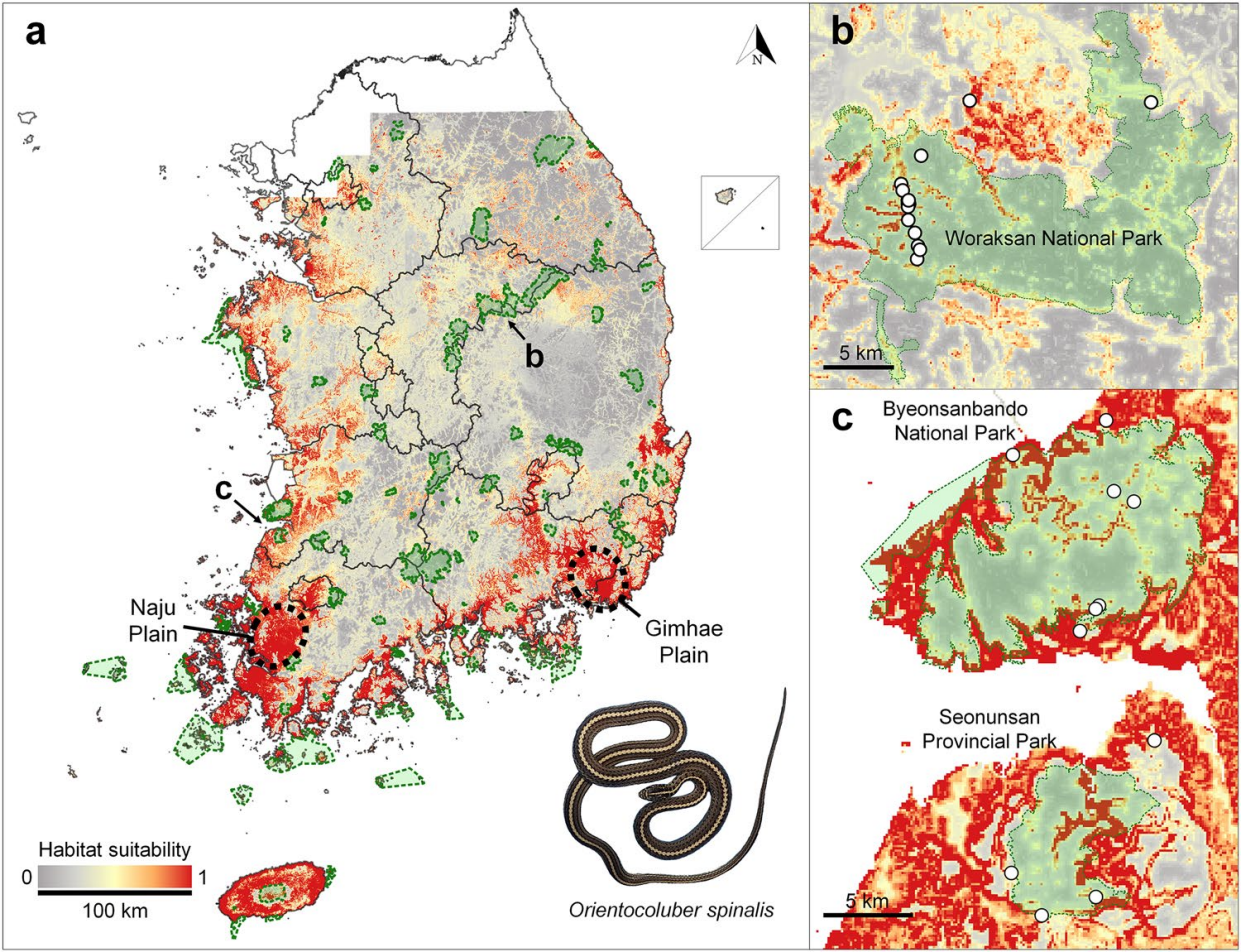
The habitat suitability for *Orientocoluber spinalis* and protected area in the Republic of Korea. Green zone indicates the three typical protected areas including national parks, provincial parks, and county parks (**a**). The red area is habitable for *O. spinalis* over the threshold. On the mainland, 23 snakes were observed in the Woraksan National Park (**b**), seven in the Byeonsanbando National Park, and four in the Seonunsan Provincial Park (**c**). White circles indicate the location data of *O. spinalis*. This map was generated using QGIS v.3.4.7 (https://www.qgis.org).

Among the three base models, random forest (RF) model showed the highest accuracy and transferability with the highest area under the receiver operating characteristic curve (AUC) and true skill statistic (TSS) values, followed by boosted regression trees (BRT, also known as the generalized boosting method/GBM) and maximum entropy (MaxEnt; Table 3). The ensemble model had the most accurate predictability with AUC of 0.966 and TSS of 0.805 compared to the three other ENMs. Further, the ensemble model indicated that altitude had the greatest contribution (34.6%), followed by the annual mean temperature (21.3%), distance to forest (20.0%), slope (13.3%), distance to grassland (5.5%), and annual precipitation (5.4%). The ensemble model predicted that *O. spinalis* had only 11,217 km^2^ as a suitable habitat area, which represented 13.5% of the total evaluated area of 83,049 km^2^ (Table 1). The habitable area for the mainland area was 8462 km^2^ (10.8%) out of 78,422 km^2^, while the island area had a habitable area of 2755 km^2^ (59.5%) out of 4627 km^2^. Habitable areas were mainly located in the western and southern coastal regions, comprising great agricultural plains and islands in the southwestern sea, while the areas were limited to the northern and eastern mainland regions (Fig. 3). In addition, habitable areas were found in several large cities, such as Gwangju, Busan, and Ulsan.

**Table 3:** Validation and explanation ability of four ecological niche models and the habitable areas above the threshold values. Habitable area is defined as an area with a suitability value above the threshold. The Percentage indicates the habitable area relative to the whole study area. AUC = area under curve; TSS = true skill statistic; RF = random forest; BRT = boosted regression tree; MaxEnt = maximum entropy.

Model	AUC	TSS	Sensitivity	Specificity	Threshold	Habitable area (km^2^)
RF	0.928	0.765	0.813	0.911	0.193	10,007 (12.0%)
BRT	0.863	0.590	0.790	0.777	0.121	14,455 (17.4%)
MaxEnt	0.838	0.579	0.758	0.752	0.491	15,354 (18.5%)
Ensemble	0.966	0.805	0.893	0.912	0.268	11,217 (13.5%)

## Discussion

The combined application of field surveys and high-resolution land cover analysis enabled us to characterize the key habitat components of *O. spinalis* and develop empirical high-resolution ENMs. The ensemble model showed the highest AUC and TSS values, providing reliable results. This approach allowed for more precise identification of the distribution pattern of *O. spinalis* and a realistic threat assessment.

The predominant land cover types surrounding the location point of *O. spinalis* were forest, grassland, and dry cropland. Among these, the closest land cover type to the location point was grassland, followed by forest and bare land. These results suggest that *O. spinalis* primarily inhabited grassland, cropland, or bare land adjoining the forest edge. Based on our field surveys and a previous radiotelemetry study^38^, *O*. *spinalis* was predominantly observed in grasslands adjacent to forests. The average altitude of the individual location data that made the greatest contribution to ENMs was 110 m a.s.l. Based on the topographical results and habitat analyses, we inferred that the preferred habitat of *O. spinalis* consisted of grassland, cropland, or bare land adjoining the edges of relatively flat lowland forests. Such habitat usage characteristics are often reported in *O. spinalis*^38^ and various other grassland snake species, including the Eastern racer (*Coluber constrictor*), Grass snake (*Natrix natrix*), and the Common garter snake (*Thamnophis sirtalis*)^39–42^.

Despite the ENM, indicating large suitable habitats for *O. spinalis* in the southwestern and southeastern parts of the Korean Peninsula, particularly in agricultural plains like the Naju and Gimhae Plains, our field observations revealed low occurrences of *O. spinalis* in these areas. We speculate that *O. spinalis* inhabits lowland adjoining forests for two possible reasons. First, anthropogenic activities can fundamentally change suitable habitats into hostile ones. Irreplaceable reptile habitats are closely related to anthropogenic pressure^43^. Most land in the plains has experienced arable land rearrangements, such that the land’s topography has greatly changed. Following paddy cultivation, the basking and feeding areas were highly disrupted. Such disturbances could also negatively influence populations of small snakes or lizards, which are the major components of *O. spinalis* diet^33,44^. Second, habitable areas for *O. spinalis* are conveniently accessible for anthropogenic activities. Since mountainous forests account for 64.5% of the total land area in the Republic of Korea^45^, anthropogenic development is largely concentrated in the lowlands. Therefore, the grassland area in the Republic of Korea decreased by approximately 50% in 2020 compared to 1995^46^. These disturbances would have led to further habitat fragmentation and destruction. Apart from agricultural plains, we documented several individuals of *O. spinalis* from protected mainland areas, such as Woraksan and Byeonsanbando National Parks and Seonunsan Provincial Park. Despite the lack of major components, including large grasslands or dry croplands, offered by key habitats, these protected areas provided suitable basking and foraging sites. In addition, even though suitable areas were found in several large urban areas, we rarely found *O. spinalis* at the outermost edge of the cities, indicating that cities were unsuitable for *O. spinalis*.

Contrary to the limited observations in mainland areas, we recorded numerous individuals of *O. spinalis* on the islands. We suppose several reasons for this observation. Firstly, many islands with small mountainous forests, adjacent grasslands, or dry croplands are highly habitable for *O. spinalis*, as shown in our models. These environments provided sufficient resources, such as basking and foraging areas for *O. spinalis*^38^. For example, the Japanese keelback (*Hebius vibakari*) and Tsushima smooth skink (*Scincella vandenburghi*), which are known prey, are enough for islands^44^. Secondly, anthropogenic activities in islands are limited owing to the low human population density compared to mainland areas. In addition, dry cropland, which can be used as an alternative for *O. spinalis* to grasslands, is used as arable land^40,47^, instead of rice paddies, which are unsuitable in small islands. Third, suitable habitats are usually limited to small islands, thereby increasing the observation chances of *O. spinalis*.

The priority of conservation efforts of either island or mainland populations of *O. spinalis* is a key question. We identified habitable areas on the islands, agricultural plains, and a few forested areas and observed many, very few, and few individuals in the order. However, despite the abundance of island populations in the southwestern sea, island populations pose several challenges in terms of long-term conservation. First, island populations are vulnerable to demographic variability^48,49^. They are isolated from each other by the sea barrier, limiting gene flow among them and often having very low genetic diversity^50,51^. In our previous study, 27 individuals on Oeyeon Island had only one haplotype for the mitochondrial *Cytb* and *ND4* genes^52^. Second, the island population could be highly vulnerable to the expected climate change, which could move the entire *O. spinalis* distribution to the north^30^. Since island populations cannot shift their latitudinal distribution range, progressive climate change could accelerate the growth of the entire island population in the Republic of Korea in the near future. Although the mainland, Woraksan, and Byeonsanbando National Parks, have more than five populations within 500 km^2^, the population density is very low. In our previous study, mainland populations, such as the Woraksan population, had relatively high genetic diversity, resulting in gene flow between subpopulations^52^. Although anthropogenic disturbances such as forest roads limit certain gene flows in mountainous mainland populations, the situation is better than that among island populations. Considering our current and previous results, we propose that for the long-term survival of *O. spinalis* in the Republic of Korea, more attention should be paid to mainland mountainous populations located in protected areas than island populations.

The mainland Republic of Korea has very few populations of *O. spinalis*, with stable populations reported only from Woraksan and Byeonsanbando National Parks and Seonunsan Provincial Park. Despite high population densities on islands, *O. spinalis* populations are isolated from each other and have low genetic diversity, with low priority for conservation purposes. Currently, the Korean *O. spinalis* population is threatened as to be designated as Endangered under the IUCN assessment criteria^53^, based on our current results on key habitats and distribution rates and previous results on low genetic diversity and large climate change impacts^30,52^. Therefore, we propose designating *O. spinalis* as an endangered species in the Republic of Korea for its long-term conservation. Future conservation efforts should be directed primarily toward valuable mainland mountainous populations, including reducing anthropogenic disturbances in the lower ranges of montane forests. Furthermore, as this is the first distribution and habitat assessment on *O. spinalis* worldwide, we expect that this approach can be effectively applied not only to the Republic of Korea, but also to other Asian countries such as Russia, Mongolia, and Kazakhstan where *O. spinalis* is also threatened.

## Methods

### Location data collection

We extracted and compiled location data for *O. spinalis* from various Korean research institutions, such as the National Institute of Ecology (*n* = 52) and the Korea National Park Service (*n* = 31); public databases, such as Inaturalist (https://www.inaturalist.org; *n* = 14) and Naturing (https://www.naturing.net; *n* = 3); and from several Korean reptile researchers’ records (*n* = 40). We then applied the following four criteria to the compiled dataset to filter out unreliable or inaccurate data points: (1) data points recorded prior to 2000, (2) inaccurate coordinates, including regional centroids (e.g., downtown) or coordinates recorded on the sea, (3) no detailed coordinate information to the fifth decimal place, and 4) data without the name of the recorder and collection date. Additionally, we screened 106 data points collected through field surveys conducted between 2020 and 2022. During the field surveys, we considered each live individual, shed skin, and carcass as a unique data point. We arranged the obtained location data based on administrative districts comprising eight metropolitan cities and nine provinces in the Republic of Korea (Table 1, Fig. 1).

Since spatial autocorrelation may occur in adjacent location data^54^, especially in the field survey sites, we removed overlapping data points within a 1 km radius and finally selected 123 location data for the analysis^30,55,56^. To select location data, we used QGIS v.3.4.7^57^.

### Habitat characteristics analysis

We considered 12 variables to characterize the habitat of *O. spinalis*: eight land cover types, two topographic variables, and two climatic variables. For land-cover types, we downloaded seven raster layers, each representing the cover of urban areas, cropland, forest, grassland, wetland, bare land, and freshwater bodies, at a 1 m × 1 m grid resolution (Korea Ministry of Environment; https://egis.me.go.kr/; Fig. 1). Considering the distinctly different ecological roles of dry cropland and rice paddy as animal habitats, we further subdivided the cropland into rice paddy and dry cropland, resulting in a final set of eight land cover types; land cover data near the Northern Limit Line (NLL; near the border between South and North Korea) were not provided due to military security reasons (Fig. 1).

To identify the important land cover types for *O. spinalis* habitat, we calculated the area of eight land cover types within a 100 m radius of each location and the distances between each location and the eight major land cover types (Table 2). We considered that the area (approximately 3.14 ha) within a 100 m radius was sufficient to represent the habitat of *O. spinalis*, as the species’ home range is approximately 2.11 ha^38^. We defined the shortest distance from the location point for each land-cover type as the distance to that land-cover type. In addition, we visualized the distribution density of snakes according to distance from each land cover type using the package ggplot2^58^ in R v. 4.0.5 software package^59^.

To extract topographic variables (altitude and slope), we downloaded numerical topographic data at a 1 m × 1 m grid resolution (National Geographic Information Institution; http://map.ngii.go.kr/). Based on the contour lines of the topographic map, we constructed a digital elevation model and extracted the altitude and slope.

For climatic variables, we selected annual mean temperature (AMT) and annual precipitation (APP), which are the most commonly used variables in distribution models implemented for reptiles^60^. We produced these climatic variables by applying the inverse distance-weighted interpolation method with average climatic data collected from 1991 to 2020 from 95 meteorological stations provided by the Korea Meteorological Administration (https://data.kma.go.kr/). We used QGIS v.3.4.7 for the generation of environmental variables^57^.

### Ecological niche modeling

#### Environmental variables

To identify and evaluate suitable habitats for *O. spinalis* in the Republic of Korea, we built ENMs using six environmental variables: distance to forest (DTF), distance to grassland (DTG), altitude, slope, AMT, and APP. Although many different environmental variables can be considered to generate ENMs, the specificity of presence data increases with increasing variables, leading to a high false negative rate^61^. Therefore, we only included the six variables with low multicollinearity (| *r* |< 0.6)^62^. We used DTF and DTG as indicators of the major habitats of the eight land-cover types. These two types constituted most of the habitat areas and were closest to the location data. A previous study also suggested that forests and grasslands are major factors in *O. spinalis* habitat^38^. Since area variables are difficult to apply in ENMs, we used only distance variables. Snakes generally prefer grasslands along forest edges and avoid deep forest interiors^39,42^; therefore, we treated the DTF as having a higher positive value further away from the forest and a higher negative value deeper in the forest interior. However, we treated DTG as having only positive and zero values because grasslands in the Republic of Korea do not have a large continuous area and are less negatively affected by depth than forests. For the remaining variables, we selected two geographic (altitude and slope) and two climatic (AMT and APP) variables because they are the most basic variables for ENMs applied to animals^60^. Owing to the heavy computational operating load with high-resolution data of 1 m × 1 m covering the Republic of Korea, we resampled the variables to a relatively lower grid resolution of 120 m × 120 m, which is the highest spatial resolution that can be handled by the biomod2 package^63^.

#### Selecting ENMs

We generated the final ENM for *O. spinalis* within the ensemble-modeling framework. The ensemble model is a multimodal approach that addresses the shortcomings of individual ENM algorithms, reinforces their strengths^63,64^, and cross-validation^60^. Ensemble methods have been frequently used to determine habitat suitability for various snake species^65,66^. The proper selection of individual base ENMs is critical for effective habitat prediction using the ensemble model. We selected random forest (RF), boosted regression trees (BRT), and maximum entropy (MaxEnt) as base models and relatively recently developed machine-learning algorithms for interpolation with high reliability^67–70^. Random forest, an approach that combines several randomized decision tree predictors of independent samples^71,72^, has been shown to have high classification accuracy and high stability against small perturbations of data^71,73^. The BRT combines many simple regression decision trees using boosting techniques, is robust to nonlinear relationships, and can process interaction effects between predictors^74,75^. MaxEnt estimates the target distribution by calculating the convergence value (maximum entropy) based on its experimental average, which enables it to incorporate interactions between predictors and calculate the optimal probability distribution^76^. ENMs were created using the package biomod2^63^ in R version 4.0.5^59^.

#### Model evaluation

After building the ENMs using three different algorithms, we combined these models into a final ensemble model weighting the true skill statistic (TSS)^77^ cutoff value of 0.6. We generated individual ENMs in 15 bootstrap replicates and 5,000 iterations, using 123 presence and 1,000 pseudo-absence data. We randomly selected 25% of the location data for the model evaluation. We used the area under the receiver operating characteristic curve (AUC) and the TSS to evaluate the predictive performance of the ENMs. The AUC suggests the optimal correlation between sensitivity and 1-specificity and is typically used to evaluate calculations based on machine learning methods^78^. The TSS was calculated as sensitivity + specificity – 1, presenting a simple and intuitive measure of ENM performance^77^. The AUC value between 0.5 and 0.7 indicates moderate predictive performance, between 0.7 and 0.9 indicates high model performance, and AUC greater than 0.9 indicates excellent model performance^79^. Meanwhile, the TSS value between 0.4 and 0.6 indicates moderate predictive performance, between 0.6 and 0.7 indicates high model performance, and TSS greater than 0.7 indicates excellent model performance^79^. We used the value maximizing the sum of sensitivity and specificity (MaxSSS)^80^ to threshold the continuous model prediction output into a binary presence-absence map. The MaxSSS threshold draws a reliable interpretation because maximizing the sum of sensitivity and specificity is equivalent to minimizing the sum of false negatives and false positives^81^. We then overlaid the distribution of protected areas in the Republic of Korea (national parks, provincial parks, and county parks; Korea Database on Protected Areas; http://www.kdpa.kr/) on the ENM to visually examine the overlap between the protected areas, location data, and suitable habitats predicted by the ENMs (Fig. 3).

## Supplementary Information

Supplementary Information.

## Data Availability

The datasets generated and analyzed in the current study are available from the corresponding author upon reasonable request.
