# Supplementary material for: Distribution and habitat assessments of the Slender racer, Orientocoluber spinalis, for the registration of nationally endangered species in the Republic of Korea
Source: Sci Rep. 2023 Jul 25;13:12025. doi: 10.1038/s41598-023-39018-4 (PMC10368646; doi:10.1038/s41598-023-39018-4)
Supplement: Supplementary file 1 — Supplementary Information. [file 41598_2023_39018_MOESM1_ESM.docx]

**Supplementary Table 1.** Contribution rates of six variables for building ecological niche models. The values are presented as the average value ± standard deviation, as each model was built with 15 replicates. RF = random forest; BRT = boosted regression tree; MaxEnt = maximum entropy.

| **Variable** | **RF** | **BRT** | **MaxEnt** | **Ensemble** |
| --- | --- | --- | --- | --- |
| Altitude | 50.3 ± 4.6 | 34.2 ± 6.7 | 39.0 ± 11.8 | 41.2 ± 6.3 |
| Annual mean temperature | 23.1 ± 2.0 | 27.2 ± 3.7 | 25.5 ± 2.3 | 25.3 ± 1.9 |
| Distance to forest | 15.3 ± 1.6 | 25.0 ± 4.8 | 25.4 ± 6.5 | 21.9 ± 3.6 |
| Slope | 18.1 ± 2.9 | 5.3 ± 1.4 | 25.2 ± 7.2 | 16.2 ± 3.3 |
| Annual precipitation | 5.3 ± 1.2 | 1.3 ± 0.5 | 13.8 ± 3.1 | 6.8 ± 1.4 |
| Distance to grassland | 8.1 ± 0.9 | 3.3 ± 1.4 | 4.9 ± 1.7 | 5.4 ± 1.0 |

**Supplementary Table 2.** Location of *Orientocoluber spinalis* in the Republic of Korea. I: island; M: mainland.

| **No.** | **Province** | **Location data (°)** | | **Collection year** | **Land type** | **Selection** |
| --- | --- | --- | --- | --- | --- | --- |
|  |  | **Latitude** | **Longitude** |  |  |  |
| 1 | JN | 34.983347 | 126.135998 | 2022 | I | ○ |
| 2 | JN | 34.608609 | 125.829947 | 2021 | I |  |
| 3 | JN | 34.610850 | 125.824142 | 2021 | I |  |
| 4 | JN | 34.603554 | 125.825340 | 2021 | I |  |
| 5 | JN | 34.603456 | 125.827501 | 2021 | I |  |
| 6 | JN | 34.612385 | 125.821105 | 2021 | I |  |
| 7 | JN | 34.613592 | 125.820943 | 2021 | I | ○ |
| 8 | JN | 34.504616 | 127.788159 | 2021 | I | ○ |
| 9 | JN | 34.610979 | 125.825437 | 2020 | I |  |
| 10 | JN | 34.602836 | 125.830001 | 2020 | I |  |
| 11 | JN | 34.300833 | 126.050833 | 2020 | I | ○ |
| 12 | JN | 34.752292 | 125.894557 | 2018 | I | ○ |
| 13 | JN | 34.608611 | 125.829167 | 2018 | I |  |
| 14 | JN | 34.292103 | 126.067572 | 2018 | I | ○ |
| 15 | JN | 34.285331 | 126.076433 | 2018 | I | ○ |
| 16 | JN | 34.752295 | 125.894535 | 2018 | I |  |
| 17 | JN | 34.608611 | 125.829167 | 2018 | I |  |
| 18 | JN | 34.602767 | 125.829767 | 2018 | I | ○ |
| 19 | JN | 33.984183 | 126.927833 | 2017 | I | ○ |
| 20 | JN | 34.448898 | 127.327742 | 2017 | M | ○ |
| 21 | JN | 34.448889 | 127.327778 | 2017 | I |  |
| 22 | JN | 34.625775 | 127.313908 | 2017 | M | ○ |
| 23 | JN | 34.465956 | 126.283055 | 2016 | I | ○ |
| 24 | JN | 34.610294 | 125.832156 | 2016 | I |  |
| 25 | JN | 35.202772 | 126.147168 | 2016 | I |  |
| 26 | JN | 34.920639 | 126.222755 | 2016 | I | ○ |
| 27 | JN | 34.349934 | 127.033872 | 2016 | I | ○ |
| 28 | JN | 35.203856 | 126.147771 | 2015 | I | ○ |
| 29 | JN | 35.208062 | 126.149468 | 2015 | I |  |
| 30 | JN | 34.700972 | 125.981056 | 2015 | I | ○ |
| 31 | JN | 34.783139 | 126.491000 | 2015 | M | ○ |
| 32 | JN | 34.737211 | 126.688258 | 2014 | M | ○ |
| 33 | JN | 34.418116 | 126.514781 | 2013 | M |  |
| 34 | JN | 34.301472 | 126.037142 | 2013 | I |  |
| 35 | JN | 34.519778 | 126.547111 | 2013 | M | ○ |
| 36 | JN | 34.540167 | 126.492083 | 2013 | M | ○ |
| 37 | JN | 34.547389 | 126.485250 | 2013 | M | ○ |
| 38 | JN | 34.420083 | 126.514194 | 2013 | M | ○ |
| 39 | JN | 34.427444 | 126.537556 | 2013 | M | ○ |
| 40 | JN | 34.301556 | 126.037528 | 2013 | I |  |
| 41 | JN | 34.297611 | 126.035833 | 2013 | I | ○ |
| 42 | JN | 34.409912 | 126.121568 | 2012 | I | ○ |
| 43 | JN | 35.348417 | 126.031536 | 2012 | I | ○ |
| 44 | JN | 34.388513 | 125.297712 | 2012 | I | ○ |
| 45 | JN | 34.800438 | 125.996027 | 2012 | I | ○ |
| 46 | JN | 34.772614 | 125.921306 | 2012 | I | ○ |
| 47 | JN | 34.777030 | 125.952479 | 2012 | I | ○ |
| 48 | JN | 34.698825 | 125.934633 | 2012 | I | ○ |
| 49 | JN | 34.903658 | 126.064169 | 2012 | I | ○ |
| 50 | JN | 34.867547 | 126.011381 | 2012 | I | ○ |
| 51 | JN | 34.865494 | 126.048672 | 2012 | I | ○ |
| 52 | JN | 34.826222 | 126.089311 | 2012 | I | ○ |
| 53 | JN | 34.761544 | 126.080547 | 2012 | I | ○ |
| 54 | JN | 34.855036 | 126.237933 | 2012 | I | ○ |
| 55 | JN | 34.727464 | 126.111369 | 2012 | I | ○ |
| 56 | JN | 34.704139 | 126.129211 | 2012 | I | ○ |
| 57 | JN | 34.703131 | 126.163525 | 2012 | I | ○ |
| 58 | JN | 34.634933 | 126.149736 | 2012 | I | ○ |
| 59 | JN | 34.553947 | 126.068153 | 2012 | I | ○ |
| 60 | JN | 34.537160 | 126.044420 | 2012 | I | ○ |
| 61 | JN | 34.180297 | 126.561842 | 2012 | I | ○ |
| 62 | JN | 34.366689 | 126.513092 | 2012 | M | ○ |
| 63 | JN | 34.428029 | 127.038482 | 2011 | I | ○ |
| 64 | JN | 34.222681 | 127.233613 | 2011 | I | ○ |
| 65 | JN | 34.241467 | 127.253742 | 2011 | I | ○ |
| 66 | JN | 34.336603 | 126.845172 | 2011 | I | ○ |
| 67 | JN | 34.323543 | 126.843349 | 2011 | I | ○ |
| 68 | JN | 34.608833 | 125.870806 | 2009 | I | ○ |
| 69 | JN | 34.916700 | 126.439392 | 2009 | M | ○ |
| 70 | JN | 34.752051 | 125.893207 | 2008 | I |  |
| 71 | JN | 34.601222 | 125.820333 | 2008 | I |  |
| 72 | JN | 34.509389 | 127.758417 | 2008 | I | ○ |
| 73 | JN | 34.465253 | 127.466606 | 2008 | I | ○ |
| 74 | JN | 34.303361 | 126.030306 | 2008 | I |  |
| 75 | JN | 34.289611 | 126.063000 | 2008 | I |  |
| 76 | JN | 34.674680 | 125.374031 | 2008 | I | ○ |
| 77 | JN | 34.247644 | 126.054456 | 2007 | I | ○ |
| 78 | JN | 35.267194 | 126.925833 | 2007 | M | ○ |
| 79 | JN | 35.294533 | 127.098024 | 2007 | M | ○ |
| 80 | CN | 36.225428 | 126.072415 | 2022 | I | ○ |
| 81 | CN | 36.233744 | 126.089430 | 2022 | I |  |
| 82 | CN | 36.225072 | 126.085662 | 2022 | I |  |
| 83 | CN | 36.229899 | 126.083975 | 2021 | I |  |
| 84 | CN | 36.229896 | 126.084101 | 2021 | I |  |
| 85 | CN | 36.230058 | 126.083091 | 2021 | I |  |
| 86 | CN | 36.230026 | 126.083079 | 2021 | I |  |
| 87 | CN | 36.231353 | 126.079459 | 2021 | I |  |
| 88 | CN | 36.230089 | 126.083798 | 2021 | I |  |
| 89 | CN | 36.228925 | 126.083065 | 2021 | I |  |
| 90 | CN | 36.231133 | 126.082237 | 2021 | I |  |
| 91 | CN | 36.230029 | 126.083713 | 2021 | I |  |
| 92 | CN | 36.229864 | 126.084159 | 2021 | I |  |
| 93 | CN | 36.230731 | 126.082510 | 2021 | I |  |
| 94 | CN | 36.229809 | 126.084400 | 2021 | I |  |
| 95 | CN | 36.229048 | 126.083161 | 2021 | I |  |
| 96 | CN | 36.231032 | 126.082009 | 2021 | I |  |
| 97 | CN | 36.230022 | 126.084520 | 2021 | I |  |
| 98 | CN | 36.230169 | 126.083123 | 2021 | I |  |
| 99 | CN | 36.229043 | 126.085186 | 2021 | I |  |
| 100 | CN | 36.230134 | 126.083713 | 2021 | I |  |
| 101 | CN | 36.230089 | 126.082714 | 2021 | I |  |
| 102 | CN | 36.230064 | 126.083536 | 2021 | I |  |
| 103 | CN | 36.229125 | 126.084702 | 2021 | I |  |
| 104 | CN | 36.228903 | 126.080935 | 2021 | I |  |
| 105 | CN | 36.228914 | 126.080766 | 2021 | I |  |
| 106 | CN | 36.228651 | 126.080793 | 2021 | I |  |
| 107 | CN | 36.677508 | 126.075511 | 2021 | I | ○ |
| 108 | CN | 36.674687 | 126.077887 | 2021 | I |  |
| 109 | CN | 36.675833 | 126.069378 | 2021 | I |  |
| 110 | CN | 36.233806 | 126.089244 | 2020 | I |  |
| 111 | CN | 36.233280 | 126.089992 | 2020 | I |  |
| 112 | CN | 36.230072 | 126.082851 | 2020 | I |  |
| 113 | CN | 36.230072 | 126.082851 | 2020 | I |  |
| 114 | CN | 36.229955 | 126.084065 | 2020 | I |  |
| 115 | CN | 36.229913 | 126.084016 | 2020 | I |  |
| 116 | CN | 36.229713 | 126.084676 | 2020 | I |  |
| 117 | CN | 36.230044 | 126.083808 | 2020 | I |  |
| 118 | CN | 36.228857 | 126.084246 | 2020 | I |  |
| 119 | CN | 36.230983 | 126.081379 | 2020 | I |  |
| 120 | CN | 36.229954 | 126.083796 | 2020 | I | ○ |
| 121 | CN | 36.230930 | 126.081692 | 2020 | I |  |
| 122 | CN | 36.228851 | 126.084491 | 2020 | I |  |
| 123 | CN | 36.229890 | 126.083768 | 2020 | I |  |
| 124 | CN | 36.229947 | 126.083953 | 2020 | I |  |
| 125 | CN | 36.229947 | 126.083953 | 2020 | I |  |
| 126 | CN | 36.232124 | 126.087707 | 2020 | I |  |
| 127 | CN | 36.230204 | 126.083812 | 2020 | I |  |
| 128 | CN | 36.230092 | 126.083652 | 2020 | I |  |
| 129 | CN | 36.229985 | 126.083753 | 2020 | I |  |
| 130 | CN | 36.233535 | 126.089003 | 2020 | I |  |
| 131 | CN | 36.230052 | 126.083713 | 2020 | I |  |
| 132 | CN | 36.230038 | 126.083026 | 2020 | I |  |
| 133 | CN | 36.228979 | 126.086336 | 2018 | I |  |
| 134 | CN | 36.850247 | 126.198724 | 2017 | M | ○ |
| 135 | CN | 36.227969 | 126.077039 | 2016 | I |  |
| 136 | CN | 36.227486 | 126.076831 | 2015 | I |  |
| 137 | CN | 36.201083 | 126.539111 | 2013 | M | ○ |
| 138 | CN | 36.405327 | 126.844625 | 2008 | M | ○ |
| 139 | JJ | 33.497251 | 126.963012 | 2022 | I |  |
| 140 | JJ | 33.497725 | 126.963056 | 2022 | I |  |
| 141 | JJ | 33.497316 | 126.962904 | 2022 | I |  |
| 142 | JJ | 33.497783 | 126.962946 | 2022 | I |  |
| 143 | JJ | 33.497742 | 126.962977 | 2022 | I |  |
| 144 | JJ | 33.497034 | 126.964524 | 2022 | I |  |
| 145 | JJ | 33.496860 | 126.964644 | 2022 | I |  |
| 146 | JJ | 33.496309 | 126.965334 | 2022 | I |  |
| 147 | JJ | 33.497055 | 126.963142 | 2022 | I |  |
| 148 | JJ | 33.493573 | 126.966076 | 2022 | I |  |
| 149 | JJ | 33.497239 | 126.962965 | 2022 | I |  |
| 150 | JJ | 33.496814 | 126.964691 | 2022 | I |  |
| 151 | JJ | 33.495706 | 126.965897 | 2022 | I |  |
| 152 | JJ | 33.497733 | 126.963045 | 2022 | I |  |
| 153 | JJ | 33.494110 | 126.958728 | 2022 | I |  |
| 154 | JJ | 33.495526 | 126.965923 | 2022 | I |  |
| 155 | JJ | 33.492521 | 126.962842 | 2022 | I |  |
| 156 | JJ | 33.497147 | 126.957046 | 2022 | I |  |
| 157 | JJ | 33.497307 | 126.963689 | 2022 | I |  |
| 158 | JJ | 33.494857 | 126.966065 | 2022 | I |  |
| 159 | JJ | 33.497177 | 126.964287 | 2022 | I |  |
| 160 | JJ | 33.494782 | 126.957936 | 2022 | I |  |
| 161 | JJ | 33.497853 | 126.960065 | 2022 | I |  |
| 162 | JJ | 33.497275 | 126.961719 | 2022 | I |  |
| 163 | JJ | 33.495912 | 126.956461 | 2022 | I |  |
| 164 | JJ | 33.363283 | 126.357029 | 2021 | I | ○ |
| 165 | JJ | 33.503875 | 126.947950 | 2021 | I |  |
| 166 | JJ | 33.200233 | 126.269982 | 2021 | I | ○ |
| 167 | JJ | 33.504329 | 126.956046 | 2021 | I | ○ |
| 168 | JJ | 33.503893 | 126.956561 | 2021 | I |  |
| 169 | JJ | 33.561239 | 126.762497 | 2020 | I | ○ |
| 170 | JJ | 33.222968 | 126.295630 | 2020 | I |  |
| 171 | JJ | 33.439608 | 126.823230 | 2018 | I | ○ |
| 172 | JJ | 33.401995 | 126.710368 | 2018 | I | ○ |
| 173 | JJ | 33.496717 | 126.962497 | 2017 | I | ○ |
| 174 | JJ | 33.427507 | 126.933900 | 2011 | I | ○ |
| 175 | JJ | 33.307325 | 126.570217 | 2011 | I | ○ |
| 176 | JJ | 33.429795 | 126.935492 | 2011 | I |  |
| 177 | JJ | 33.424301 | 126.527000 | 2009 | I | ○ |
| 178 | JJ | 33.357278 | 126.306389 | 2009 | I | ○ |
| 179 | JJ | 33.203111 | 126.290500 | 2009 | I | ○ |
| 180 | CB | 36.864136 | 128.088019 | 2021 | M |  |
| 181 | CB | 36.863836 | 128.087898 | 2021 | M | ○ |
| 182 | CB | 36.863836 | 128.087898 | 2021 | I |  |
| 183 | CB | 36.863628 | 128.088633 | 2020 | M |  |
| 184 | CB | 36.863902 | 128.088060 | 2020 | M |  |
| 185 | CB | 36.863912 | 128.087917 | 2020 | M |  |
| 186 | CB | 36.863878 | 128.088548 | 2020 | M |  |
| 187 | CB | 36.876381 | 128.082500 | 2020 | M | ○ |
| 188 | CB | 36.836888 | 128.095271 | 2017 | M |  |
| 189 | CB | 36.834472 | 128.096778 | 2017 | M | ○ |
| 190 | CB | 36.863922 | 128.087675 | 2013 | M |  |
| 191 | CB | 36.926358 | 128.281286 | 2013 | M | ○ |
| 192 | CB | 36.928878 | 128.137572 | 2013 | M | ○ |
| 193 | CB | 36.862228 | 128.087806 | 2013 | M |  |
| 194 | CB | 36.894265 | 128.098592 | 2013 | M | ○ |
| 195 | CB | 36.515281 | 127.856286 | 2013 | M | ○ |
| 196 | CB | 36.828556 | 128.094444 | 2009 | M |  |
| 197 | CB | 36.861917 | 128.087750 | 2009 | M |  |
| 198 | CB | 36.872313 | 128.083649 | 2009 | M |  |
| 199 | CB | 36.866000 | 128.088220 | 2009 | M |  |
| 200 | CB | 36.853472 | 128.087806 | 2009 | M | ○ |
| 201 | CB | 36.866004 | 128.087618 | 2008 | M |  |
| 202 | CB | 36.864778 | 128.088222 | 2008 | M |  |
| 203 | CB | 36.845242 | 128.092914 | 2008 | M | ○ |
| 204 | JB | 35.466399 | 126.600545 | 2020 | M | ○ |
| 205 | JB | 35.614065 | 126.601738 | 2018 | M |  |
| 206 | JB | 35.690229 | 126.547815 | 2018 | M | ○ |
| 207 | JB | 35.672099 | 126.610737 | 2018 | M | ○ |
| 208 | JB | 35.601141 | 126.590006 | 2017 | M | ○ |
| 209 | JB | 35.612389 | 126.599667 | 2017 | M | ○ |
| 210 | JB | 35.707994 | 126.605725 | 2015 | M | ○ |
| 211 | JB | 35.456953 | 126.567014 | 2014 | M | ○ |
| 212 | JB | 35.478381 | 126.548089 | 2013 | M | ○ |
| 213 | JB | 35.666862 | 126.623189 | 2010 | M | ○ |
| 214 | JB | 35.545833 | 126.636513 | 2006 | M | ○ |
| 215 | GB | 36.698845 | 128.131907 | 2020 | M | ○ |
| 216 | GB | 35.730342 | 128.416553 | 2020 | M | ○ |
| 217 | GB | 36.502430 | 128.054040 | 2019 | M | ○ |
| 218 | GB | 36.770470 | 128.814750 | 2016 | M | ○ |
| 219 | GB | 36.718542 | 128.090726 | 2014 | M | ○ |
| 220 | GB | 36.130389 | 128.695669 | 2011 | M | ○ |
| 221 | GB | 36.877667 | 129.156397 | 2007 | M | ○ |
| 222 | GB | 36.873696 | 129.160712 | 2007 | M |  |
| 223 | GW | 37.165173 | 128.341732 | 2016 | M | ○ |
| 224 | GW | 37.211583 | 128.422611 | 2014 | M | ○ |
| 225 | GW | 37.166974 | 128.342602 | 2011 | M |  |
| 226 | GW | 37.739333 | 127.687667 | 2009 | M | ○ |
| 227 | GW | 37.764222 | 128.577720 | 2008 | M | ○ |
| 228 | GW | 37.740583 | 128.404500 | 2006 | M | ○ |
| 229 | GW | 37.764271 | 128.578340 | 2006 | M |  |
| 230 | GN | 34.666555 | 128.251059 | 2022 | I | ○ |
| 231 | GN | 35.562936 | 128.123531 | 2013 | M | ○ |
| 232 | GN | 35.414561 | 128.870295 | 2008 | M | ○ |
| 233 | GN | 35.526923 | 127.831732 | 2006 | M | ○ |
| 234 | GN | 35.563308 | 127.914502 | 2006 | M | ○ |
| 235 | IC | 37.080642 | 125.947051 | 2014 | I | ○ |
| 236 | IC | 37.266306 | 126.480573 | 2008 | I | ○ |
| 237 | IC | 37.249292 | 126.315628 | 2003 | I | ○ |
| 238 | IC | 37.271967 | 126.470733 | 2002 | I | ○ |
| 239 | IC | 37.379067 | 126.429739 | 2001 | I | ○ |
| 240 | GG | 37.080642 | 126.947051 | 2014 | M |  |
| 241 | GG | 37.567583 | 127.798917 | 2009 | M | ○ |
| 242 | GG | 37.080525 | 126.947133 | 2008 | M | ○ |
| 243 | GG | 37.849917 | 127.288971 | 2008 | M | ○ |
| 244 | DG | 35.776600 | 128.421249 | 2014 | M | ○ |
| 245 | DG | 35.777028 | 128.585381 | 2014 | M | ○ |
| 246 | SU | 37.555364 | 126.874763 | 2021 | M | ○ |

**
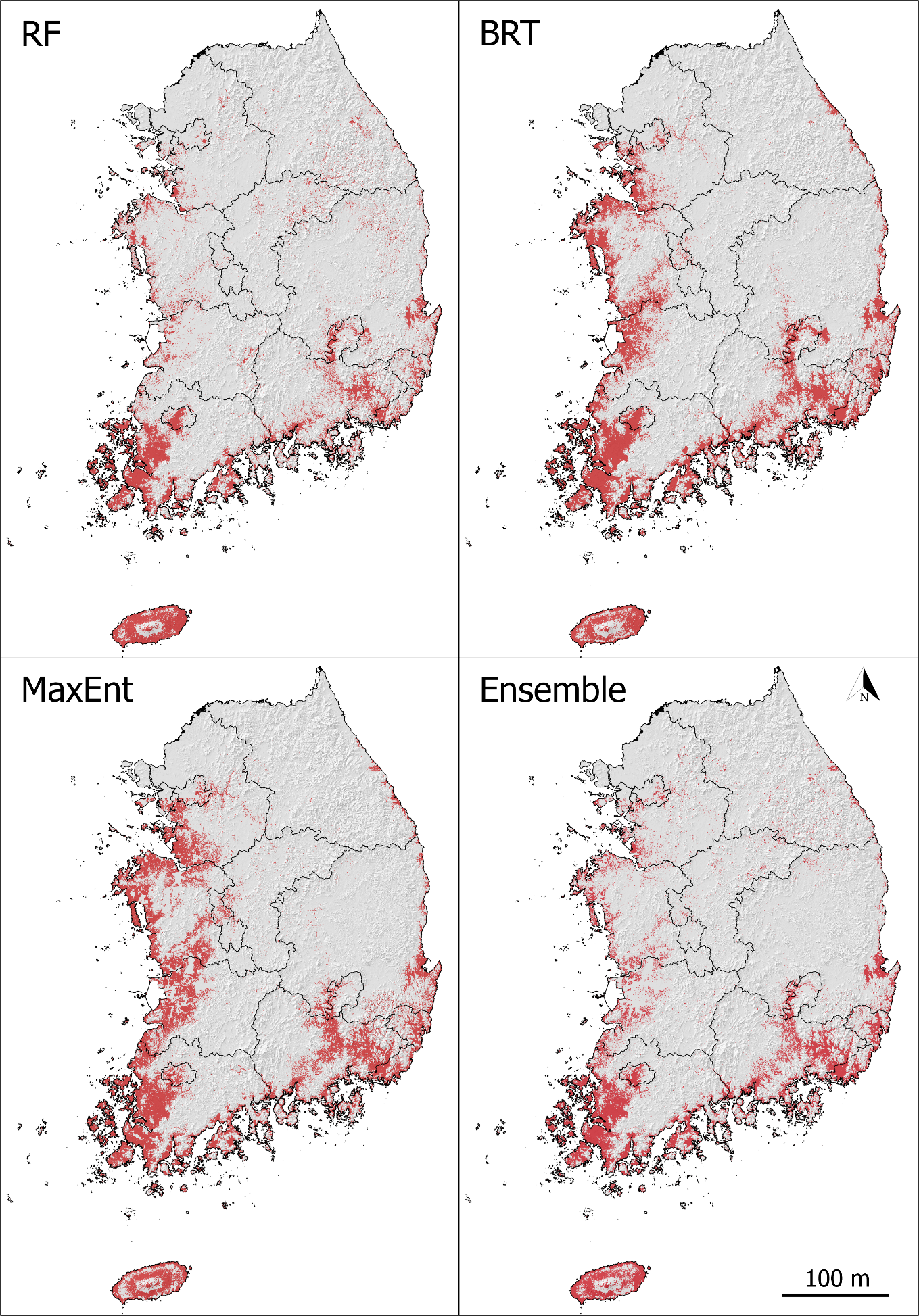
**

Supplementary Figure 1. Distribution of habitable areas in four ecological niche models. The red and gray parts indicate habitable areas calculated above the threshold value and areas unsuitable for *Orientocoluber spinalis* habitat, respectively. RF: random forest, BRT: boosted regression trees, and MaxEnt: maximum entropy. This map was generated using QGIS v.3.4.7 (https://www.qgis.org).
